# Development of intestinal microflora and occurrence of diarrhoea in sucking foals: effects of *Bacillus cereus* var*. toyoi* supplementation

**DOI:** 10.1186/s12917-015-0355-3

**Published:** 2015-02-14

**Authors:** Jenny John, Kathrin Roediger, Wieland Schroedl, Nada Aldaher, Ingrid Vervuert

**Affiliations:** Institute of Animal Nutrition, Nutrition Diseases and Dietetics, Faculty of Veterinary Medicine, University of Leipzig, Leipzig, Germany; Pferdeklinik Großostheim, Großostheim, Germany; Institute of Bacteriology and Mycology, Faculty of Veterinary Medicine, University of Leipzig, Leipzig, Germany; Present address: Tierklinik Teisendorf, Teisendorf, Germany

**Keywords:** Microflora, Foal, Diarrhoea, Probiotic, *Bacillus cereus* var. *toyoi*

## Abstract

**Background:**

Almost all foals develop transient diarrhoea within the first weeks of life. Studies indicated different viral, bacterial, and parasitic causes, such as rotavirus, *Clostridium perfringens*, *Escherichia coli,* and *Cryptosporidium* are discussed. But little is known about the development of intestinal microflora in foals. The present study investigated whether the supplementation with *Bacillus cereus* var. *toyoi* would modify the developing intestinal microflora and consequently reduce diarrhoea in foals. From birth, the foals were randomly assigned to three treatment groups: placebo (10 mL isotonic NaCl, *n* = 8), low dosage (LD; 5 × 10^8^ cfu *B. cereus* var*. toyoi*, *n* = 7) and high dosage (HD; 2 × 10^9^ cfu *B. cereus* var*. toyoi, n* = 10). Treatment groups were supplemented orally once a day for 58 days. Faeces scoring and sampling were performed within the first 24 h after birth and on day 9, 16, 23, 30, 44, 58 of the foal’s life and also on the first day of diarrhoea. Culture-plate methods were used to analyse the bacterial microflora.

**Results:**

Eighty-eight per cent of the foals developed diarrhoea (placebo 7/8, LD 5/7, HD 10/10) during the first 58 days of life. *Bacillus cereus* var. *toyoi* supplementation had no effect on bacterial microflora. *Clostridium perfringens* and enterobacteria were equally prevalent in foals with diarrhoea and those who were not afflicted.

**Conclusions:**

We conclude that the supplementation of *B. cereus* var. *toyoi* had no effect on the occurrence of diarrhoea and health status in the foals.

**Electronic supplementary material:**

The online version of this article (doi:10.1186/s12917-015-0355-3) contains supplementary material, which is available to authorized users.

## Background

Diarrhoea is a common problem in neonatal foals. Almost all foals develop transient diarrhoea within the first weeks of life [1,2]. Studies have reported different viral, bacterial, and parasitic causes [3–6]. Between the days 5 and 15 of a foal’s life, when the dam’s first post partum oestrus is expected, diarrhoea in foals is observed frequently [3]. The diarrhoea is termed ‘foal heat diarrhoea’. Although commonly there is no reduction in behaviour of the foals, some of them suffer from diarrhoea more than 20 days within the first 2 months of life and develop not as good as foals with shorter diarrhoea periods. The establishment of intestinal microflora and maturation of the gastrointestinal mucosa are some reasons proposed for diarrhoea in this period of life [2]. However, little is known about the development of the intestinal microflora during this period. During and following birth, neonates are exposed to a variety of microorganisms originally from the dam or environment. On the day of birth, aerobes, facultative anaerobes, and strict anaerobes were already detected in the faeces of the foals [7]. Before foals are fed solid food, cellulolytic bacteria have already colonized the digestive tract [7]. Because the intestinal microflora is a crucial line of resistance against colonization by exogenous microbes, it is highly relevant in the prevention of tissue invasion by pathogens [8].

Probiotic bacteria were defined by a joint FAO, WHO [9] as ‘live microorganism which when administered in adequate amounts confer a health benefit on the host’. Some probiotics are known to have a positive effect on the intestinal microflora. For example, Yuyama et al. [10] reported that supplementation with probiotics led to an earlier recovery from foal heat diarrhoea, probably by enhancing establishment of the normal intestinal microflora. However, other studies that showed that supplementation with probiotics in foals increased the occurrence and duration of diarrhoea compared to placebo-treated foals [11], and Weese and Rousseau [12] reported that probiotic administration was significantly associated with development of signs of depression, anorexia, and colic in foals.

The aim of the study was to investigate the effects of oral probiotic supplementation on the growing intestinal microflora in equine neonates. Previous studies have shown positive effects of *Bacillus cereus* var. *toyoi* on intestinal health in calves, piglets, poultry, broiler chickens, and growing rabbits [13–16]. Baum et al. [17] observed trophic effects of *Bacillus cereus* var. toyoi on the small intestinal mucosa of pigs as demonstrated by longer villi, thicker mucosa and more mature, i.e. acidic mucins. Vilà et al. [15] showed that feeding of *Bacillus cereus* var. *toyoi* reduced the prevalence of pathogens such as Salmonella in poultry and improved the performance variables of broiler chickens. We hypothesized that oral supplementation with *B. cereus* var. *toyoi* from birth until 2 months of the foal’s life would modify the intestinal microflora and mucosa, and therefore lead to a reduction of diarrhoea in foals.

## Results

During the observation period, haematolgy parameters like erythrocytes, haematocrit or leukocytes were in the reference range obtained for foals (data not shown, treatment p > 0.05). 24 h after birth serum IgG levels were always higher than the lowest critical threshold of 8 g/L. Serum IgG levels of the foals were in median between 17.1 g/l and 25.6 g/l (treatment p > 0.05). During the observation period, serum IgG levels decreased down to 60 – 70% of initial value at day 58 (time p < 0.05, treatment p > 0.05). Diarrhoea occurred in up to 90% of the foals for at least one 1 day between days 8 and 16 of life. In particular, during transition period between orange-brown milk faeces and more adult-like greenish faeces a loose consistency (faeces score: 6–9) was observed in 92% of the foals. Despite this, foals remained bright and alert and continued to nurse.

Supplementation with *B. cereus* var. *toyoi* had no significant effect on dry matter content of the faeces of the foals. Furthermore, supplementation with *B. cereus* var. *toyoi* had no significant effect on blood parameters and there was no change in the incidence of diarrhoea in foals in the first 58 days of life (Table 1).

**Table 1:** **Number of foals with diarrhoea, duration of diarrhoea (days) and faecal score in placebo (** ***n*** **= 8), low dosage (** ***n*** **= 7), and high dosage (** ***n*** **= 10) during period of oral supplementation with** ***Bacillus cereus*** **var.** ***toyoi*** **(days 1 to 58 of life), data expressed as numbers, median, 25 and 75% quartile**

**Item**	**Placebo**	**Low dosage**	**High dosage**	**P-value**
**Number of foals with diarrhoea (n)**				
	7/8	5/7	10/10	>0.05
**Duration of diarrhoea (days)**				
25% quartile	2	2	2.8	0.7072
Median	2.5	10	3.5	
75% quartile	5.3	11	4.8	
**Faecal score**				
25% quartile	3	2	2	0.132
Median	3	3	3	
75% quartile	3	6	5	

Lactobacilli in the foals faeces increased on days 1 to 9 of foals life. Maximum level was 4.50 × 10^7^ cfu/g (LD, Table 2). Declining counts of Lactobacilli were noticed until day 58 where Lactobacilli counts almost reached the levels of the mares (measured at the time of birth). Supplementation with *B. cereus* var. *toyoi* had no significant effect on lactobacilli counts in faeces of the foals.

**Table 2:** **Lactobacilli in faeces (cfu/g) of the foals from days 1 to 58 of life in placebo, low dosage (LD), and high dosage (HD) groups and for all foals, data expressed as numbers, median, 25 and 75% quartile, time p = 0.002, treatment p = 0.522**

**Group**	**Measure**	**Day of foal’s life**							
		**1**	**3**	**9**	**16**	**23**	**30**	**44**	**58**
**Placebo**	25% quartile	2.8 × 10^4^	2.7 × 10^6^	3.2 × 10^6^	2.1 × 10^6^	2.9 × 10^5^	5.0 × 10^5^	4.3 × 10^5^	4.1 × 10^4^
	Median	3.4 × 10^4^	1.7 × 10^7^	2.5 × 10^7^	2.0 × 10^7^	1.4 × 10^6^	1.0 × 10^6^	1.9 × 10^6^	2.2 × 10^5^
	75% quartile	4.0 × 10^5^	8.3 × 10^7^	4.0 × 10^7^	3.0 × 10^7^	5.0 × 10^6^	2.5 × 10^6^	2.6 × 10^6^	3.3 × 10^5^
**LD**	25% quartile	1.9 × 10^4^	n.d.	8.2 × 10^6^	3.6 × 10^6^	2.5 × 10^5^	2.8 × 10^5^	3.4 × 10^5^	2.7 × 10^5^
	Median	4.0 × 10^5^	n.d.	4.5 × 10^7^	5.5 × 10^6^	5.4 × 10^5^	3.9 × 10^5^	4.0 × 10^5^	6.0 × 10^5^
	75% quartile	2.0 × 10^6^	n.d.	6.7 × 10^7^	1.9 × 10^7^	2.9 × 10^6^	4.2 × 10^5^	5.4 × 10^5^	9.7 × 10^5^
**HD**	25% quartile	7.0 × 10^3^	2.3 × 10^6^	4.7 × 10^6^	4.2 × 10^6^	2.7 × 10^5^	3.1 × 10^5^	1.4 × 10^5^	1.5 × 10^5^
	Median	2.5 × 10^4^	3.7 × 10^6^	2.1 × 10^7^	1.5 × 10^7^	1.6 × 10^6^	2.7 × 10^6^	4.3 × 10^5^	4.9 × 10^5^
	75% quartile	2.3 × 10^6^	3.8 × 10^7^	4.6 × 10^7^	4.6 × 10^7^	1.3 × 10^7^	7.1 × 10^6^	7.3 × 10^5^	4.4 × 10^6^
**Total**	25% quartile	1.6 × 10^4^	2.3 × 10^6^	3.5 × 10^6^	3.2 × 10^6^	1.9 × 10^5^	2.5 × 10^5^	2.7 × 10^5^	1.9 × 10^5^
	Median	3.4 × 10^4^	3.7 × 10^6^	2.3 × 10^7^	1.7 × 10^6^	1.3 × 10^6^	6.0 × 10^5^	5.0 × 10^5^	3.2 × 10^5^
	75% quartile	2.2 × 10^6^	4.5 × 10^7^	5.0 × 10^7^	2.8 × 10^7^	5.0 × 10^6^	2.9 × 10^6^	1.2 × 10^6^	1.0 × 10^6^

n.d.: non-determinable, total: over all treatments.

Both frequency of detection (Table 3) and bacteria counts of enterococci (data not shown) in the faeces of the foals increased in the first 9 days of life and then declined until day 58. Enterococci were found in 15.2% of the mares samples (measured at the time of birth). Supplementation with *B. cereus* var. *toyoi* had no significant effect on enterococci counts in faeces of the foals.

**Table 3:** **Detection rate (> 10** ^**3**^ **cfu/g,%) of enterococci in faeces of the foals from days 1 to 58 of life in placebo, low dosage (LD), and high dosage (HD) groups and for all foals, data expressed in %, treatment p > 0.05**

**Group**	**Day of foal’s life**							
	**1**	**3**	**9**	**16**	**23**	**30**	**44**	**58**
**Placebo**	66,7%	100.0%	85.7%	50.0%	50.0%	25.0%	25.0%	12.5%
**LD**	57.1%	n.d.	85.7%	85.7%	42.9%	42.9%	42.9%	14.3%
**HD**	60.0%	83.3%	100.0%	60.0%	40.0%	40.0%	40.0%	30.0%
**Total**	61.1%	90.0%	91.7%	64.0%	44.0%	36.0%	36.0%	20.0%

n.d.: non-determinable, total: over all treatments.

*Bacteroides spp*. were detected in the faeces of three foals respectively in one to three samples between days 9 and 30 of life. Two out of three foals had days of diarrhoea above-the average. Four out of six samples with *Bacteroides spp*. were samples from diarrhoea. No *Bacteroides spp*. were found in the mares samples (data not shown).

Enterobacteria were not detected in faeces of the foals at every time point during the treatment period (days 1 to 58 of life, Table 4). Enterobacteria were found in 30% (10 of 33) of the mare samples (measured at the time of birth), at a maximum of 6.00 × 10^5^ cfu/g (data not shown). Enterobacteria were equally prevalent in foals with diarrhoea and in foals those not afflicted (data not shown). No significant treatment effect was observed for enterobacteria.

**Table 4:** **Detection rate (> 10** ^**3**^ **cfu/g,%) of enterobacteria in faeces of the from days 1 to 58 of life in placebo, low dosage (LD), and high dosage (HD) groups and for all foals, data expressed in %, treatment p > 0.05**

**Group**	**Day of foal’s life**							
	**1**	**3**	**9**	**16**	**23**	**30**	**44**	**58**
**Placebo**	0%	25.0%	14.3%	25.0%	25.0%	37.5%	37.5%	50.0%
**LD**	28.6%	n.d.	42.9%	42.9%	42.9%	57.1%	28.6%	57.1%
**HD**	20.0%	16.7%	60.0%	40.0%	40.0%	30.0%	40.0%	20.0%
**Total**	16.7%	20.0%	36.0%	36.0%	40.0%	40.0%	36.0%	40.0%

n.d.: non-determinable, total: over all treatments.

In the first faeces of the foals after meconium, *Clostridium perfringens* was detectable in 50–71% of the samples (Table 5). In the placebo group, *C. perfringens* was detectable in 100% of the foals on day 3. Among all groups, the maximum count was 6.00 × 10^6^ cfu/g on day 3. *C. perfringens* was found in 75% of the mare samples (measured at the time of birth) and ranged from 1.00 × 10^3^ to 2.50 × 10^5^ cfu/g (data not shown). *Clostridium perfringens* was equally prevalent in foals with or without diarrhoea (data not shown). Supplementation with *B. cereus* var. *toyoi* had no significant effect on *C. perfringens*.

**Table 5:** **Detection rate (> 10** ^**3**^ **cfu/g,%) of** ***Clostridium perfringens*** **in faeces of the foals from days 1 to 58 of life in placebo, low dosage (LD), and high dosage (HD) groups and for all, foals, data expressed in %, treatment p > 0.05**

**Group**	**Day of foal’s life**							
	**1**	**3**	**9**	**16**	**23**	**30**	**44**	**58**
**Placebo**	50.0%	100%	42.9%	25.0%	12.5%	0%	0%	0%
**LD**	71.4%	n.d.	71.4%	71.4%	28.6%	14.3%	14.3%	28.6%
**HD**	60.0%	66.7%	90.0%	60.0%	10.0%	0%	0%	0%
**Total**	61.1%	80.0%	70.8%	52.0%	16.0%	4.0%	4.0%	8.0%

n.d.: non-determinable, total: over all treatments.

Occasionally yeasts were found in samples of the mares (2 out of 24 samples) and the foals (17 out of 191 samples, data not shown).

## Discussion

The present study was performed on a studfarm under typical field conditions. To minimize effects related to husbandry, feeding, and season, our study took place at one thoroughbred farm, within one foaling period from February to May. According to Lahrssen and Zentek [18] such a study design is important when working with a limited number of animals. Probiotic dosing and treatment period were in accordance with studies by Jeroch et al. [19], Jadamus et al. [20], and Vilà et al. [15].

Unfortunately some foals were treated with antibiotics during a severe period of illness which may have an impact on the subsequent microbial profile in faeces.

Bacterial microflora in faeces was used as an indication of the effects of *B. cereus* var. *toyoi* on the intestinal health of foals. Although faeces might have some limitations for describing the gut ecosystem, by comparing microflora and the digestion process in the colon with faeces in fistulated horses Julliand and Goachet [21] showed that the faecal ecosystem is an appropriate marker of intestinal changes appearing in the colon ecosystem.

Culture-plate methods were used to access bacterial microflora in the faeces of the foals to establish basic knowledge. For following studies culture-dependent methods should be extended by PCR methods to improve knowledge about diversity of genes.

In our study, diarrhoea did not lead to changes in normal foal behaviour. Foals remained bright and alert and continued to nurse. Clinical parameters including heart and respiratory rate, body temperature, and body weight were in the proper physiological range. We conclude that diarrhoea in foals between days 6 and 16 of the foal’s life is not primarily pathogen related. During this period, we observed diarrhoea when orange-brownish faeces changed to green, soft faeces. These changes in faeces colour might mark the transition from only digestion of milk to increasing digestion of solid nutrients like crude fibre. In that context, hemicellulose and cellulose could be responsible for a higher water-binding capacity and a reduction of intestinal passage. As a result, adsorptive and secretory intestinal processes are influenced and free water in the colon could simulate lower dry matter content in faeces of the foals. Another reason for the reduction of dry matter content in faeces could be osmotically-acting metabolites of bacterial digestion of crude fibre.

Meconium has been reported to be free of bacteria [22] and no bacterial PCR products were obtained from meconium samples [11], which confirmed that the gastrointestinal tract of a foetus is sterile [23]. In mares, bacterial flora remained largely stable from 14 days before until 42 days after foaling, whereas the presence of bacterial species and intensity of bacterial growth changed over time in foals [2]. These results were confirmed in our study, as aerobic and anaerobic bacteria (data not shown), but also lactobacilli, enterococci and *C. perfringens* counts in the faeces of the foals increased after birth until day 3 or day 9. Foals start to consume forages and concentrates very early in life. As a result, the intestinal microflora is adapting rapidly to improve digestion of the feed. In foals, there may be great genetic selective pressure for early colonisation of microflora to avoid acidic stomach conditions and to occupy a more distal region (post-gastric) than would be the case in calves or lambs [24]. By day 58 of a foal’s life, aerobic and anaerobic bacteria, enterobacteria, lactobacilli, and enterococci had decreased to the levels in the faeces of mares (measured at the time of birth). These changes seemed to be related to the stabilisation of microflora by increasing fibre intake and digestion processes within the first 2 months of the foal’s life.

*Bacillus cereus* var. *toyoi* did not have any effects on the health status of the foals. There is a great diversity of bacteriophages, bacteria, fungi, and protozoa that might have an unselective entry to the gastrointestinal tract of the foals. Therefore, even if probiotic bacteria survive the acidic conditions after day 2 of life, they will experience heavy competition within the very rapidly developing intestinal microflora of foals.

Probiotic supplementation with *Lactobacillus pentosus* WE7 was significantly associated with development of signs of depression, anorexia, and colic in foals [12]. Also, supplementation with *Lactobacillus rhamnosus* and *Enterococcus faecium* led to increased diarrhoea in foals [11]. In contrast, we found that supplementation with *B. cereus* var. *toyoi* had no effect on the growth and health parameters in foals kept under high-quality standard husbandry.

## Conclusions

Supplementation with *B. cereus* var. *toyoi* had no effect on health status or the intestinal microflora in suckling foals. Diarrhoea occurred in up to 90% of the foals for at least one 1 day between days 8 and 16 of life. Despite this, foals remained bright and alert and continued to nurse. Diarrhoea might be a part of the normal physiological development of the intestinal microflora. Competition with diverse, rapidly colonizing intestinal microflora seems to suppress *B. cereus* var. *toyoi* and its possible effects.

## Methods

### Animals

The project (V54-19c20/15-V/04) was approved by the Ethics Committee for Animal Rights Protection of the District government in Darmstadt, in accordance with German legislation for animal rights and welfare.

A total of 25 mares and their foals with one thoroughbred stud were included in the study. The mares had been stabled at least 2 months before foaling at the stud farm. Mares were kept in separate stalls with straw beddings and were turned out daily on sand paddocks for several hours every day. They were fed ad libitum haylage or hay and 2–3 kg of concentrates per day. Mares had free access to fresh water at all times. Foals were born between February and May 2011. Twenty-two foals first came into contact with the mares’ udder within the first hour of life, and 1 foal required 90 min. (In the other two mares, foaling was not observed.) Mares were dewormed with ivermectin immediately after foaling and 2–3 months post partum. After foaling, mares were fed ad libitum hay, 4–5 kg of alfalfa hay, and 2–3 kg of a complement feed per day (Equilac Zuchtstutenfutter Spezial Etzean; Additional file 1: Table S1). Passive transfer of antibodies was controlled with SNAP Foal IgG Test (Idexx Laboratories, Westbrook, Maine, USA). Twenty-four of the 25 foals had immunoglobulin levels of > 800 mg/dL at 12 h of age.

### Feeding

In the first weeks of life foals had access to the mare’s feed. From day 2 of the foal’s life, the mare and foal were turned out on sand paddocks twice a day for 1–2 h. From day 7 to 14, two mares and foals had access to pasture twice a day for 2 to 4 h. At this time, no additional alfalfa hay was fed to the mares. From 4 to 8 weeks, groups of mares and foals were turned out on pasture and foals were fed complement feed for foals (1.7–2.5 kg per foal; Fohlenfutter Spezial Etzean; Additional file 1: Table S1) in a separate area. Once or twice a week foals were given 50 mL of a mineral mixture (Meganutril junior; Additional file 1: Table S1).

### Deworming and treatments

All foals received pyrantel in week 2 of life, ivermectin in week 8, and pyrantel in month 4. Five foals were treated for an omphalic inflammation with cefquinome or trimethoprim-sulfadimethoxine at day 2–4 or at day 24–26 of foals life, two foals were treated for congenital neuromyodysplasia with oxytetracycline at day 2–4 of foals life and three foals were treated because of high-grade diarrhoea with cefquinome or trimethoprim-sulfadimethoxine at day 24–26 of foals life.

### Probiotic and administration protocol

*Bacillus cereus* var. *toyoi* was provided as an odourless, white to greyish-brown dry powder supplemented with 1.0 × 10^10^ viable spores of *B. cereus* var. *toyoi* per gram. The viability was confirmed at the beginning of the study by use of the microbiological spread plate method.

Foals were randomly allocated to three groups and received their respective placebo or supplement once a day orally for 58 days, starting on the first day of life. Eight foals were given 10 mL of isotonic saline solution (placebo), seven foals received 5.0 × 10^8^ cfu of *B. cereus* var. *toyoi* (low dosage, LD), and ten foals were given 2.0 × 10^9^ cfu of *B. cereus* var. *toyoi* (high dosage, HD).

### Examinations and faeces samples

Behaviour, appetite, heart and respiratory rates, rectal temperature, and body weights were monitored during the treatment period (day 1 to 58 of life) and monthly from age 3 to 5 months. Blood work was done four times in month 1 and at the end of month 2. The time schedule of the study is given in Table 6.

**Table 6:** **Time schedule of the study**

**Measure**	**Day of foal’s life**												**Month of foal’s life**		
	**1**	**4**	**6**	9*	**10**	**12**	**14**	**16**	**23**	**30**	**44**	**58**	**3**	**4**	**5**
Health status	X	X	X	X	X	X	X	X	X	X	X	X	X	X	X
Body weight	X			X				X	X	X	X	X	X	X	X
Faeces	X			X				X	X	X	X	X			
Blood	X			X				X		X		X			

*Additional measurements and sampling were done on the first day of diarrhoea.

Within the first day of life, on days 9, 16, 23, 30, 44, and 58, and on the first day of diarrhoea faecal samples were taken from the rectum or by the use of a collection bag. Faeces were visually classified by a faeces score (Table 7), and faeces dry matter content was determined after oven-drying (103°C) to constant mass.

**Table 7:** **Faeces score**

**Score**	**Faeces quality**
0	Orange-brownish first faeces after meconium
1	Dry balled
2	Soft balled, formed
3	Not formed, soft
4	Pasty
5	Mushy
6	Inhomogeneous: structured and watery part
7	Mucous like
8	Green diarrhoea
9	Yellow diarrhoea

Mares’ faeces were taken from the rectum immediately after foaling. Faeces samples were frozen at −18°C until analysis.

#### Haematology and serum chemistry analysis

Serum and EDTA blood was subjected to haematology and serum chemistry determination. The parameters of red blood count and differential leukocyte count were determined photometric, optical or calculated using an ADVIA®120 haematology system (Fa. Bayer Vital GmbH, Fernwald, Germany).

Serum-IgG was determined quantitatively with a competitive ELISA as previously described by Schroedl et al. [25]. Thereby serum-IgG in the samples and equine IgG conjugated to peroxidase (purity according to SDS-PAGE > 90%) competed for antibodies against equine IgG. After washing and the addition of a chromogenic substrate quantity of peroxidase was optically measurable at 405 nm (reference wavelength 492 nm). Equine reference serum was used as standard. Out of the measuring data IgG concentration was calculated with Table Curve 2D v4 (Systat Software Inc, Chicago, USA). Limit of detection with a serum dilution factor of 4000 was 0.15 g/l. The level of specificity was at 100%.

### Bacterial analysis

Faecal specimens (0.5 g in 4.5 mL phosphate buffered saline) were serially diluted in phosphate buffered saline for quantitative bacterial investigations. Dilutions were tested for total aerobic cell numbers developing on sheep blood agar (Oxoid, Germany), gram negative cell numbers on Gassner agar (SIFIN, Berlin), Enterococci on Citrate azide tween carbonate agar (CATC agar, SIFIN, Berlin), total anaerobe cell numbers on sheep blood agar (OXOID, Germany), Lactobacilli on deMan, Rogosa and Sharpe Lactobacillus agar (MRSA agar, SIFIN, Berlin), Bacteroides spp. on sheep blood agar supplemented with vitamin K, Clostridium perfringens on sheep blood agar containing polymyxin B and neomycin, and yeasts and fungi on Sabouraud agar (SIFIN, Berlin). The total aerobic cell numbers, gram negative cell numbers and enterococci were cultured aerobically at 37 _C for 24 h. The anaerobic bacteria were cultured at 37 _C for 48 h in anaerobic chamber (MACS anaerobic workstation, Don Whitley Scientific limited, England). Yeasts and fungi were cultured aerobically at 37 _C for 5 days. The different isolated strains were identified based on their characteristic colony- and micromorphology and by their biochemical characteristics according to Bisping and Amtsberg [26] and finally analyzed with the use of matrix-assisted laser desorption/ionization time of flight (MALDI-TOF) as described by Shehata et al. [27].

### Statistical analysis

Data are expressed as median, 25% quartile, and 75% quartile. Data analysis was performed by the Statistica software program (StatSoft, Hamburg, Germany). The Shapiro-Wilk test was used to assess data for normality. Data were not normally distributed. Non-parametric tests (Kruskal-Wallis ANOVA) were used to compare the effects of time and diet. Pearson’s chi-squared test was used to compare the frequency of foals with diarrhoea between treatments.

Statistical significance was accepted at *P* < 0.05.

## Additional file

Additional file 1: Table S1. Nutrient composition of the feedstuffs provided to the foals and mares during experimental period (expressed in %).

## Abbreviations

*B. cereus* var. *toyoi*: *Bacillus cereus* var*. toyoi*
*C. perfringens*: *Clostridium perfringens*
HD: High dosage (5.0 × 10^8^ cfu of *B. cereus* var. *toyoi*)
LD: Low dosage (2.0 × 10^9^ cfu of *B. cereus* var. *toyoi*)
