# Supplementary material for: Development of intestinal microflora and occurrence of diarrhoea in sucking foals: effects of Bacillus cereus var. toyoi supplementation
Source: BMC Vet Res. 2015 Feb 14;11:34. doi: 10.1186/s12917-015-0355-3 (PMC4333172; doi:10.1186/s12917-015-0355-3)
Supplement: Additional file 1: Table S1. — Nutrient composition of the feedstuffs provided to the foals and mares during experimental period (expressed in %). [file 12917_2015_355_MOESM1_ESM.doc]

**Additional file 1. Nutrient composition of the feedstuffs provided to the foals and mares during experimental period (expressed in %)**

| **Contents** | **Complement feed for foals^1^** | **Commercial mineral mixture^2^** | **Complement feed for brood mares^3^** |
| --- | --- | --- | --- |
| Crude protein (%) | 14.0 | 0.45 | 11.2 |
| Crude fibre (%) | 8.5 | 0.0 | 13.0 |
| Crude ash (%) | 11.5 | 37.9 | 11.5 |
| Crude fat (%) | 14.0 | 0.2 | 6.0 |
| Ca (%) | 2.3 | 10.8 | 2.0 |
| P (%) | 0.7 | 9.9 | 0.5 |
| Mg (%) | 0.5 | 1.0 | 0.35 |
| Na (%) | 0.4 | 0.05 | 0.4 |
| Lysine (%) | 0.5 | 0.0 | 0.5 |
| Methionine (%) | 0.3 | 0.0 | 0.3 |

^1^ - Fohlenfutter Spezial Etzean, Mühle Ebert Dielheim GmbH, Dielheim, Germany

^2^ - Meganutril Junior, Equinavet GmbH Münster, Germany

^3^ - Equilac Zuchtstutenfutter Spezial Etzean, Mühle Ebert Dielheim GmbH, Dielheim, Germany
